# A Comprehensive Assessment of the Shared Genetic Architecture between Myopia and Open-Angle Glaucoma

**DOI:** 10.1016/j.xops.2026.101328

**Published:** 2026-07-22

**Authors:** Victor A. de Vries, Samantha S. Lee, Kelsey V. Stuart, Alicia Schulze, Michael L. Hunter, Susanne Hopf, Norbert Pfeiffer, Gareth Lingham, Robert N. Luben, Ya Xing Wang, Annechien E.G. Haarman, Milly S. Tedja, Virginie J.M. Verhoeven, Jost B. Jonas, Alexander K. Schuster, Anthony P. Khawaja, Caroline C.W. Klaver, David A. Mackey, Wishal D. Ramdas, International Glaucoma Genetics Consortium, Adriana I. Iglesias Gonzalez, Alex W. Hewitt, Alexander K. Schuster, Andrew J. Lotery, Angela J. Cree, Anthony P. Khawaja, Ayellet V. Segrè, Bryan Ang, Calvin P. Pang, Caroline Hayward, Caroline C.W. Klaver, Ching-Yu Cheng, Chiea Chuen Khor, Christopher R. Hammond, Cornelia van Duijn, David A. Mackey, Einer Stefansson, Eranga N. Vithana, Francesca Pasutto, Fridbert Jonansson, Gudmar Thorleifsson, Jacyline Koh, James F. Wilson, Jamie E. Craig, Janey L. Wiggs, Joëlle Vergroesen, John H. Fingert, Joni A. Turunen, Jost B. Jonas, Kathryn P. Burdon, Kelsey R. Brandt, Li Jia Chen, Louis R. Pasquale, Michael Kass, Nicola Cronbach, Nomdo M. Jansonius, Norbert Pfeiffer, Ozren Polašek, Paul J. Foster, Paul Mitchell, Pieter W.M. Bonnemaijer, Pirro G. Hysi, Puya Gharahkhani, Robert Wojciechowski, Sjoerd Driessen, Stuart MacGregor, Stuart Tompson, Terri L. Young, Tien Y. Wong, Tin Aung, Unnur Thorsteinsdottir, Victor A. de Vries, Veronique Vitart, Ananth C. Viswanathan, Wishal D. Ramdas

**Affiliations:** 1 Department of Ophthalmology, Erasmus Medical Center, Rotterdam, the Netherlands; 2 Department of Epidemiology, Erasmus Medical Center, Rotterdam, the Netherlands; 3 Centre for Ophthalmology and Visual Science (incorporating Lions Eye Institute), University of Western Australia, Perth, Australia; 4 School of Optometry and Vision Science, UNSW, Sydney, New South Wales, Australia; 5 NIHR Biomedical Research Centre, Moorfields Eye Hospital NHS Foundation Trust, and UCL Institute of Ophthalmology, London, United Kingdom; 6 Institute of Medical Biostatistics, Epidemiology and Informatics (IMBEI), University Medical Center of the Johannes Gutenberg University Mainz, Mainz, Germany; 7 School of Population and Global Health, University of Western Australia, Perth, Australia; 8 Busselton Health Study Centre, Busselton Population Medical Research Institute, Busselton, Australia; 9 Department of Ophthalmology, University Medical Center of the Johannes Gutenberg University Mainz, Mainz, Germany; 10 Centre for Eye Research Ireland, Environmental Sustainability and Health Institute, Technological University Dublin, Dublin, Ireland; 11 MRC Epidemiology Unit, University of Cambridge, Cambridge, United Kingdom; 12 Beijing Visual Science and Translational Eye Research Institute (BERI), Beijing Tsinghua Changgung Hospital, Beijing, China; 13 Tsinghua Medicine, Tsinghua University, Beijing, China; 14 Clinical Genetics, Erasmus Medical Center, Rotterdam, the Netherlands; 15 Singapore Eye Research Institute, Singapore National Eye Centre, Singapore, Singapore; 16 Department of Ophthalmology, Rothschild Foundation Hospital, Paris, France; 17 Department of Ophthalmology, Radboud University Medical Center, Nijmegen, the Netherlands; 18 Institute of Molecular and Clinical Ophthalmology, University of Basel, Basel, Switzerland

**Keywords:** Open-angle glaucoma, Myopia, Axial length, Genetic risk score, Mendelian randomization

## Abstract

**Objective:**

Individuals with high myopia have an increased prevalence of open-angle glaucoma (OAG). We aim to clarify the possibly shared genetic architecture of myopia and OAG, in particular in high myopes with myopic macular degeneration (MMD), where OAG screening is highly challenging.

**Design:**

Individual participant data meta-analysis of one-sample Mendelian randomization analyses and pleiotropic analysis under a composite null hypothesis.

**Participants:**

A total of 34 825 participants from 6 population-based cohort studies and 1 high myopia case–control study, including 708 OAG and 1953 high-myopia cases.

**Methods:**

First, we calculated and validated genetic risk scores (GRSs) for OAG and myopia in each cohort. We subsequently meta-analyzed linear and logistic regression models for the association of a myopia GRS with OAG, intraocular pressure (IOP), and vertical cup-to-disc ratio (VCDR), and the association of an OAG-GRS with high myopia, axial length, and spherical equivalent. We stratified the analysis of OAG in different stages of axial elongation, and in high myopes with or without MMD. Pleiotropic analysis under a composite null hypothesis was applied to genome-wide association study summary statistics.

**Main Outcome Measures:**

Odds ratio (OR) of OAG and high myopia, and mean difference in IOP, VCDR, axial length, and spherical equivalent.

**Results:**

One standard deviation (SD) increase in myopia GRS was associated with an OR (95% CI) of 1.18 (1.09, 1.28) for OAG, a beta (95% CI) of 0.04 (0.00, 0.08) mmHg in IOP, and of 0.005 (0.003, 0.007) in VCDR. The OAG-GRS was not significantly associated with high myopia compared to emmetropes, but a 1 SD increase was associated with a beta (95% CI) of 0.05 (0.01, 0.08) mm in axial length and of –0.05 (–0.10, –0.00) diopters in spherical equivalent. One SD increase in OAG-GRS had a substantially larger effect on OAG in high myopes with MMD, with an OR (95% CI) of 3.83 (1.89, 7.78) compared to 1.55 (1.24, 1.94) in emmetropes. Finally, we identified 95 independent pleiotropic single-nucleotide polymorphisms (SNPs).

**Conclusions:**

There is strong evidence for pleiotropy between myopia and OAG. Further research into the biological mechanisms of the identified pleiotropic SNPs is needed. An OAG-GRS might help to clinically estimate OAG risk, in particular in individuals with MMD.

**Financial Disclosure(s):**

Proprietary or commercial disclosure may be found in the Footnotes and Disclosures at the end of this article.

The prevalence of glaucoma, the leading cause of blindness worldwide, is expected to continue to increase further in the coming decades, especially the rate of open-angle glaucoma (OAG) in patients with myopia.^1^ Myopia, also known as short-sightedness, is the most common form of refractive error. Myopia currently affects approximately 1.4 billion people (almost a quarter of the world population), and this is expected to increase to 5 billion by 2050 (half of the world population), with 1 billion anticipated to have high myopia, which is typically defined as a refractive error of –6 diopters (D) or worse.^2^ Highly myopic patients, especially those with myopic macular degeneration (MMD), are a challenging group to screen and monitor for OAG. Visual field tests could be influenced by patchy or macular atrophy showing scotoma, and morphological measures often show artefacts. However, myopia is associated with a 1.6 times increased risk for OAG, and moderate/high myopia is associated with a 3.0 times increased risk.^3^

Several possible underlying mechanisms have been theorized. Most notably, connective tissue remodeling is seen in both conditions.^4^ The elongation of the eye in myopia could also increase the risk for mechanical insult of retinal ganglion cells, or inversely, increased intraocular pressure (IOP) might elongate the ocular axis, although the exact causal sequence remains unknown.^5^ Both conditions are regarded as complex polygenetic traits, with hundreds of genetic variants identified to be associated with either OAG or myopia.^6^^,^^7^ Pleiotropy is the phenomenon where a genetic variant is associated with multiple traits. Increasing evidence suggests that pleiotropy is very common among complex polygenic traits, especially cancers, autoimmune disorders, and most notably, neurodegenerative conditions.^8^, ^9^, ^10^ Given this reality and the previously reported association between myopia and OAG, it seems likely that there is substantial genetic pleiotropy between these 2 conditions. Identifying and studying the shared genetics between myopia and OAG is crucial, as it could provide new insights into the underlying pathobiology and reveal novel opportunities for early recognition of OAG in high myopes, which could improve prognosis.^11^^,^^12^

We therefore assessed the genetic overlap between myopia and OAG. We utilized data from 7 different prospective cohort studies from the International Glaucoma Genetics Consortium (IGGC) and leveraged both conventional genome-wide genetic risk score (GRS) based methods, as well as a novel single-variant approach, to reach more conclusive answers to this question.

## Methods

### Study Population

We performed an individual participant data meta-analysis of studies from the IGGC, a collaborative global initiative with the aim of promoting and facilitating the discovery of novel glaucoma genetic variants, developing genetic prediction tools, and enhancing our understanding of glaucoma etiology.^6^^,^^13^^,^^14^ To participate, studies had to have at least data available on age, sex, single nucleotide polymorphism (SNP) array data, OAG status, and axial length, or, if axial length was not available, spherical equivalent with lens status (i.e., phakic or pseudophakic). In total, 7 studies participated; full details of each contributing study are available in Table 1, the Supplementary Methods, and previous publications.^15^, ^16^, ^17^, ^18^, ^19^, ^20^, ^21^ The majority of participating studies were population-based cohort studies of middle-aged to elderly individuals. Exceptions were the Raine Study, which is a population-based birth cohort study, and the Dutch Myopia Study (MYST), which is a case–control study for high myopia.^19^^,^^21^

**Table 1:** Baseline Characteristics of Included Studies, Presented as the Mean ± Standard Deviation Unless Stated Otherwise

Trait	EPIC-Norfolk Eye Study	Gutenberg Health Study	Rotterdam Study	Busselton Healthy Aging Study	The Raine Study Gen2	Beijing Eye Study	Dutch Myopia Study
Number of participants	6164	12 389	8517	4562	1097	1154	942
Age (years)	68.7 ± 7.9	54.8 ± 10.9	62.3 ± 7.8	57.7 ± 5.72	20.4 ± 1.9	59.1 ± 9.4	41.6 ± 24.9
Sex, female (*n*, %)	3356 (54.5)	5972 (48.2)	4795 (56.3)	2479 (54.3)	479 (49.0)	719 (62.3)	550 (58.5)
IOP (mmHg)	16.3 ± 3.6	14.2 ± 2.9	13.9 ± 3.1	14.9 ± 4.1	15.3 ± 3.5	15.6 ± 2.9	14.5 ± 3.1
Adjusted IOP (mmHg)∗	16.6 ± 4.0	14.3 ± 3.2	14.2 ± 3.8	15.0 ± 4.2	N/A	15.7 ± 3.1	14.8 ± 3.7
VCDR (median, IQR)	0.4 (0.2, 0.6)	0.2 (0.1, 0.4)	0.3 (0.2, 0.4)	0.2 (0.1, 0.3)	N/A	N/A	0.3 (0.2, 0.4)
OAG (*n*, %)	217 (3.5)	93 (0.8)	252 (3.0)	84 (1.8)	N/A	35 (3.0)	27 (2.9)
Axial length (mm)	23.6 ± 1.2	23.7 ± 1.3	23.6 ± 1.3	N/A	23.6 ± 0.9	23.2 ± 1.2	26.1 ± 2.4
Spherical equivalent (diopters)	0.2 ± 2.3	–0.4 ± 2.5	0.5 ± 2.7	–0.2 ± 1.8	–0.1 ± 1.6	0.3 ± 2.14	–6.0 ± 5.7
High myopia (*n*, %)	271 (4.4)	642 (5.2)	371 (4.4)	91 (2.0)	25 (2.3)	39 (3.4)	514 (54.6)
Myopic macular degeneration (*n*, %)	N/A	40 (0.3)	89 (1.0)	9 (0.2)	N/A	3 (0.3)	105 (11.2)

EPIC = European Prospective Investigation into Cancer and Nutrition; IOP = intraocular pressure; VCDR = vertical cup-to-disc ratio; IQR = interquartile range; OAG = open-angle glaucoma; GRS = genetic risk score.

∗ Adjusted for IOP-lowering medications (IOP divided by 0.7) and IOP-lowering surgery (IOP set to 30 mmHg if IOP was below 30 mmHg prior to adjustment).

Both eyes of included participants were examined according to study-specific protocols and guidelines. Examinations consisted of varying combinations of structural and functional optic nerve head and anterior drainage angle assessments (Supplementary Methods, available at www.ophthalmologyscience.org). When multiple definitions of OAG were available, a definition based on visual field testing was preferred. Low myopia was defined as an axial length of ≥24.0 mm and <26.0 mm, or a spherical equivalent of ≤ –0.5 D and > –6.0 D if axial length was not available (excluding pseudophakic participants and participants with a history of refractive surgery). High myopia was defined as an axial length of ≥ 26.0 mm, or if axial length was not available, a spherical equivalent of ≤ –6.0 D (again excluding pseudophakic participants and participants with a history of refractive surgery). Myopic macular degeneration was graded according to the meta-analysis for pathologic myopia criteria.^22^ A meta-analysis for pathologic myopia of category 2 or higher, or the presence of a “plus” lesion (choroidal neovascularization, Fuchs spot, or lacquer cracks), was considered MMD. Participants were classified as having high myopia or OAG if at least 1 eye was diagnosed with high myopia or OAG, respectively. For continuous measures such as IOP, vertical cup-to-disc ratio (VCDR), and axial length, a random eye was chosen (or the glaucomatous eye if only 1 eye was affected). Participants of the nonmajority ancestry were excluded (European for all studies except Beijing Eye Study, where East Asians were the majority ancestry), as determined from genome-wide association study (GWAS)-data using either principal components or software packages such as Admixture (maximum likelihood for any specific ancestry >0.5).

All studies adhered to the tenets of the Declaration of Helsinki and obtained approval from their relevant institutional ethical committee (Supplementary Methods). All participants provided written informed consent before participation.

### Development of Genetic Risk Scores

We calculated a GRS for myopia and OAG in all participating cohorts by extracting the significant SNPs and corresponding betas from the most recent and largest GWAS for both eye disorders (415 SNPs for the myopia-GRS and 127 SNPs for the OAG-GRS).^6^^,^^7^ We then multiplied these betas with the dosage values (or binary genotypes if dosage values were not available) of the corresponding SNP, and the sum of these multiplications was considered the GRS for any given study participant. Most participating studies had previously carried out quality control processes for their genetic data. As a minimum standard, the following exclusions were applied: missing rate >0.03 (per person and per SNP), related individuals with pi-hat >0.2, and ancestry outliers (principal component 1 and 2 > 6 standard deviations [SDs] from mean) before imputation. Imputation was carried out using the Haplotype Reference Consortium version 1.1 reference panel, 1000 Genomes Phase 3, or the TopMed reference panel.^23^, ^24^, ^25^ Study specific details are provided in the Supplemental Methods.

### Statistical Analyses

Both the myopia-GRS and the OAG-GRS were validated within each cohort by performing a logistic regression of the GRS with its own phenotype as the outcome, as well as calculating the area under the receiver-operatoing characteristic curve (AUC). For the one-sample Mendelian randomization analyses, we tested the association of the myopia GRS on OAG, IOP, and VCDR, and the association of an OAG-GRS on high myopia, axial length, and spherical equivalent. We utilized multivariable binary logistic regression for dichotomous outcomes and linear regression models for continuous outcomes. All analyses were adjusted for age and sex. To additionally test if the association of the myopia GRS with OAG prevalence was independent of axial length, we repeated that model after matching the OAG cases with controls on axial length with a 1:3 case:control ratio. As most studies only had IOP data at the time of examination and not the initial presenting (untreated) IOP, we repeated the analysis of the myopia GRS with IOP after imputing an estimated untreated IOP. If the participant used IOP-lowering medication, the measured IOP was divided by 0.7; if the participant had undergone IOP-lowering surgery and the IOP was below 30 mmHg (after adjustment for IOP-lowering medication usage), the IOP was set to 30 mmHg.^26^^,^^27^ The results of the individual studies were meta-analyzed using a generic inverse variance-weighted approach. Heterogeneity of the meta-analyses was measured by calculating *I*^*2*^. In case of low heterogeneity (*I*^*2*^ < 50%), a fixed effects model was used in our meta-analyses. In cases of high heterogeneity (*I*^*2*^ > 50%), a random effects model was used.^28^ To evaluate the effects of both GRS on OAG in different stages of axial elongation (i.e. emmetropia vs. low myopia vs. high myopia) and in high myopia with or without MMD, we stratified the cohorts on stage of axial length and MMD status, and repeated the binary logistic regression with OAG as the outcome for both the OAG-GRS and the myopia GRS as the exposure. Only the Rotterdam Study and Dutch MYST were included in this meta-analysis. Unfortunately, MMD data were not (completely) available for the EPIC-Norfolk Study and Beijing Eye Study, not relevant for the Raine Study (mean ± SD age of 20.4 ± 1.9 years), there were insufficient numbers of MMD cases in the Busselton Healthy Aging Study for reasonably stable estimates (n = 9 cases), and only 1 participant with MMD did not have OAG in the Gutenberg Health Study.

Finally, to estimate which SNPs specifically were pleiotropic, we used pleiotropic analysis under a composite null hypothesis (PLACO), a powerful novel method for single-variant pleiotropy detection.^29^ Pleiotropic analysis under a composite null hypothesis is a robust analysis of pleiotropy that remains valid for correlated phenotypes such as myopia and OAG, even when using GWAS summary statistics from overlapping samples.^29^ Given the well-known association of myopia with OAG, we decorrelated the z-scores as implemented in PLACO before running the pleiotropy analysis. Single nucleotide polymorphisms that reached the genome-wide significance level (p < 5E-8) were considered pleiotropic SNPs. Statistically significant SNPs were clumped within a single locus if the r^2^ > 0.1 or the genomic distance was < 500 kb. Functional mapping and annotation of GWAS were used to determine the genomic regions of these pleiotropic loci and to assess potential enrichment of these genes in particular pathways, focusing on functional–annotation gene sets (from the Gene Ontology Consortium).^30^^,^^31^ Analyses were conducted with R software version 4.2.3 (R Foundation for Statistical Computing) using the tidyverse and meta packages, as well as the PLACO R program (https://github.com/RayDebashree/PLACO).

## Results

A total of 34 825 participants from 7 different studies were included, of whom 708 had OAG and 1953 were high myopes (Table 1, Table S2 available at www.ophthalmologyscience.org). Of the included studies, 5 were prospective population-based cohort studies focused on age-related conditions in middle-aged and elderly individuals, with a mean age ranging from 55.0 to 68.7 years. Conversely, the Raine Study included participants in early adulthood with a mean ± SD age of 20.4 ± 1.9 (Table 1), and the Dutch MYST included participants from a wide age range (mean ± SD 41.6 ± 24.9 years).

### Validation of Genetic Risk Scores

The association of the myopia GRS with high myopia was strong and consistent for all studies except for the Beijing Eye Study, with odds ratios (ORs) for high myopia per SD increase in myopia GRS ranging from 1.75 to 2.23, and AUCs ranging between 0.66 and 0.69 (Table S3, available at www.ophthalmologyscience.org). In the Raine Study, the only birth cohort, we found a particularly strong effect estimate, with an OR (95% confidence interval [CI]) of 2.71 (1.80, 4.10) for each SD increase in GRS and an AUC (95% CI) of 0.77 (0.67, 0.87). There was no significant association between the myopia GRS and high myopia in the Beijing Eye Study (the only Asian ancestry cohort). Thus, the Beijing Eye Study was excluded from any analyses involving the myopia GRS.

The association of the OAG-GRS with OAG was strong and consistent for all studies without exception, although the effect estimate was the smallest in the Beijing Eye Study, with an OR (95% CI) for each SD increase of 1.51 (1.06, 2.14) (Table S4, available at www.ophthalmologyscience.org). The ORs for the other included studies ranged between 1.64 and 2.16, with AUCs ranging from 0.65 to 0.74.

### Association of a Myopia Genetic Risk Score with Open-Angle Glaucoma, Intraocular Pressure, and Vertical Cup-to-Disc Ratio

**Figure 1:**
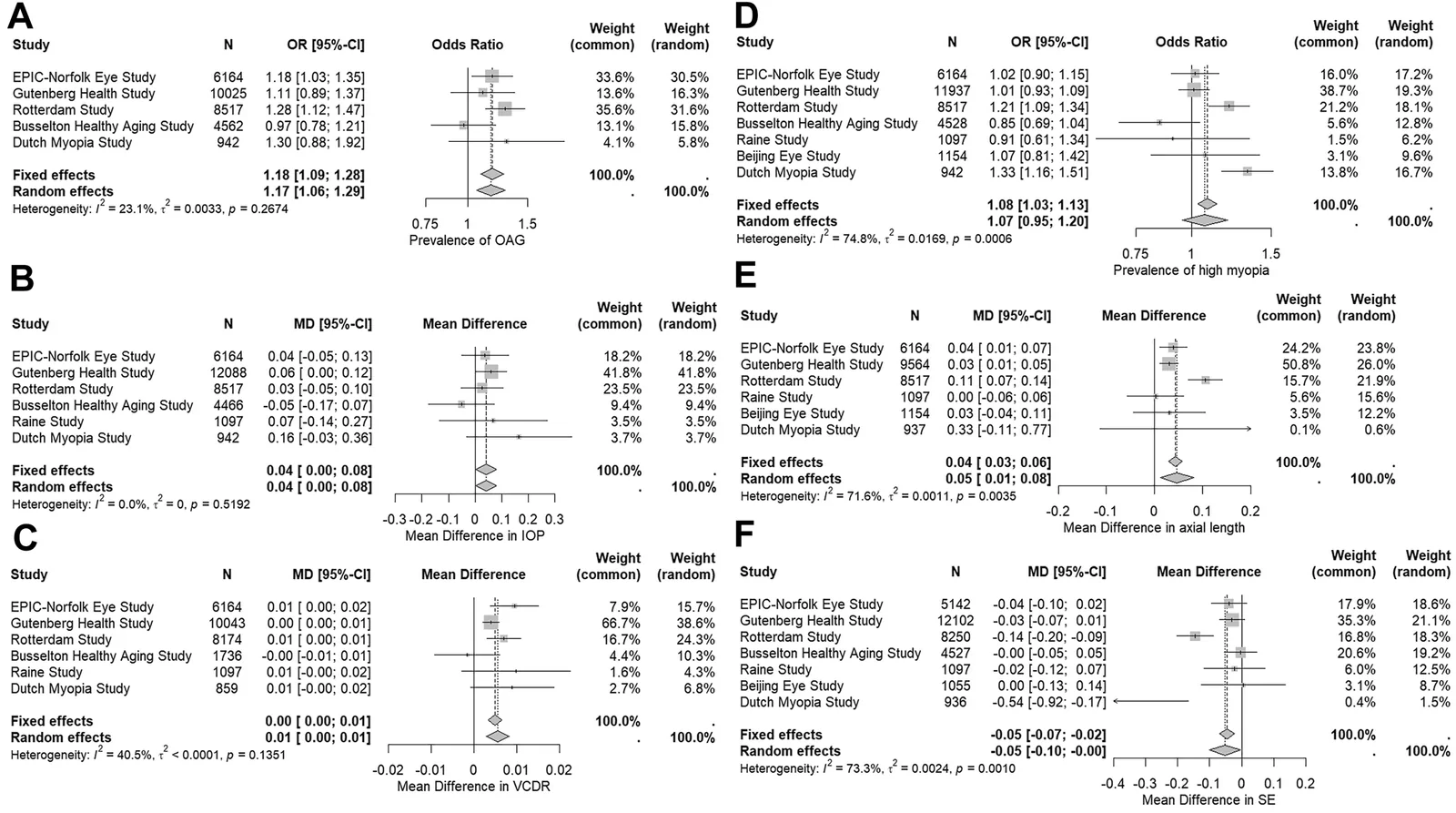
Forest plots of the meta-analyzed logistic and linear regression of a myopia GRS with OAG (**A**), IOP (**B**), and VCDR (**C**), and an OAG GRS with high myopia (**D**), axial length (**E**), spherical equivalent (**F**). Presented as the effect of a standard deviation increase in GRS. All associations were corrected for age and sex. GRS = genetic risk score, OAG = open-angle glaucoma, IOP = intraocular pressure, VCDR = vertical cup-to-disc ratio, OR = odds ratio, EPIC = European Prospective Investigation into Cancer and Nutrition, MD = mean difference.

We would like to denote here explicitly that the PLACO analyses described later demonstrate extensive pleiotropy, violating the exclusion restriction assumption required for Mendelian Randomization. These analyses should therefore be interpreted as genetic overlap analyses, not investigations of causality. One SD increase in the myopia GRS was associated with a statistically significant OR (95% CI) of 1.18 (1.09, 1.28) for OAG (Table 5, Fig 1A). This meta-analysis had relatively low heterogeneity (I^2^ = 23.1%), with most studies finding an OR between 1.11 and 1.30 (Fig 1A). Participants in the second quartile of the myopia GRS had an OR (95% CI) of 1.28 (1.01, 1.63) compared to the first quartile, whereas participants in the fourth quartile had an OR (95% CI) of 1.35 (1.06, 1.72) (Table 5) compared to the first quartile. After matching controls on axial length, the effect estimate became slightly larger but was no longer statistically significant, with an OR (95% CI) of 1.22 (0.96, 1.53). However, the second and third quartiles were significantly associated with an OR (95% CI) of 1.50 (1.12, 2.01) and 1.38 (1.00, 1.90), respectively, compared to the first quartile. Heterogeneity was also markedly higher (I^2^ = 75.2%). The myopia GRS was also associated with a statistically significant average increase (95% CI) of 0.04 (0.00, 0.08) mmHg in IOP for each SD increase in myopia GRS (Table 5, Fig 1B). After correcting the IOP for IOP-lowering medications and surgery, this effect became substantially more pronounced, with an average increase (95% CI) of 0.15 (0.01, 0.29) mmHg per SD increase in myopia GRS. Finally, a 1 SD increase in myopia GRS was also associated with a higher VCDR, with a beta (95% CI) of 0.005 (0.003, 0.007) (Fig 1C, Table 5).

**Table 5:** The Meta-Analyzed Logistic and Linear Regression of a Myopia GRS With OAG, IOP, and VCDR, and an OAG GRS With High Myopia, Axial Length, and Spherical Equivalent

Myopia GRS	Total N	Continuous	Q1	Q2	Q3	Q4	*p*-trend
OAG, OR (95% CI)	30 210	1.18 (1.09, 1.28)∗	1.0	1.28 (1.01, 1.63)∗	1.20 (0.96, 1.55)	1.35 (1.06, 1.72)∗	0.145
OAG, matched†, OR (95% CI)	2499	1.22 (0.96, 1.53)	1.0	1.50 (1.12, 2.01)∗	1.38 (1.00, 1.90)∗	1.48 (0.77, 2.86)	0.182
IOP, beta (95% CI)	33 274	0.04 (0.00, 0.08)∗	0.0	0.11 (0.02, 0.21)∗	0.06 (–0.03, 0.15)	0.13 (0.03, 0.22)∗	0.259
IOP, adjusted‡, beta (95% CI)	33 274	0.15 (0.01, 0.29)∗	0.0	0.11 (0.01, 0.21)∗	0.08 (–0.02, 0.18)	0.17 (0.03, 0.31)∗	0.160
VCDR, beta (95% CI)	28 073	0.005 (0.003, 0.007)∗	0.0	0.002 (–0.003, 0.008)	0.007 (0.002, 0.012)∗	0.013 (0.007, 0.018)∗	0.021∗

OAGGRS	Total N	Continuous	Q1	Q2	Q3	Q4	*p*-trend
High myopia, OR (95% CI)	34 339	1.07 (0.95, 1.20)	1.0	1.13 (0.99, 1.30)	1.20 (0.92, 1.56)	1.22 (0.88, 1.67)	0.074
Axial length, beta (95% CI)	27 433	0.05 (0.01, 0.08)∗	0.0	0.03 (–0.01, 0.07)	0.07 (0.00, 0.13)∗	0.17 (0.03, 0.31)∗	0.049∗
Spherical equivalent, beta (95% CI)	33 109	–0.13 (–0.27, 0.00)∗	0.0	–0.02 (–0.09, 0.05)	–0.05 (–0.12, 0.01)	–0.08 (–0.15, –0.01)∗	0.004∗

GRS = genetic risk score; OAG = open-angle glaucoma; IOP = intraocular pressure; VCDR = vertical cup-to-disc ratio; Q = quartile; OR = odds ratio; CI = confidence interval.

Presented as the effect of 1 standard deviation increase in GRS (continuous) and stratified into quartiles, with the lowest quartile as the reference category. All associations were corrected for age and sex.

∗ *P* < 0.05.

† Matched on axial length with a 1:3 case:control ratio.

‡ Adjusted for IOP-lowering medications (IOP divided by 0.7) and IOP-lowering surgery (IOP set to 30 mmHg if IOP was below 30 mmHg prior to adjustment).

### Association of an Open-Angle Glaucoma-GRS with Axial Length, Spherical Equivalent, and High Myopia

Although the OAG-GRS was not significantly associated with high myopia, it was significantly associated with axial length and spherical equivalent (Table 5). One SD increase in OAG-GRS was associated with an average increase (95% CI) of 0.05 (0.01, 0.08) mm in axial length and an average decrease (95% CI) of –0.05 (–0.10, –0.00) diopters in spherical equivalent (Fig 1E, F). Heterogeneity was high for the OAG-GRS meta-analyses, with I^2^s between 71.8% and 74.8% (Fig 1). The Dutch MYST and Beijing Eye Study were the most likely sources of heterogeneity, as the Dutch MYST was a case–control study rather than a population-based study, and the Beijing Eye Study included predominantly participants of East Asian ancestry versus European ancestry in the other studies. After exclusion of both these studies, previously significant associations remained statistically significant (Table S6, available at www.ophthalmologyscience.org), although the heterogeneity was only partially attenuated, with I^2^’s of 0.0% for spherical equivalent and high myopia, but still 77.6% for axial length.

### The Effects of Genetic Risk Score on Open-Angle Glaucoma within Stages of Axial Elongation and Myopic Macular Degeneration

**Figure 2:**
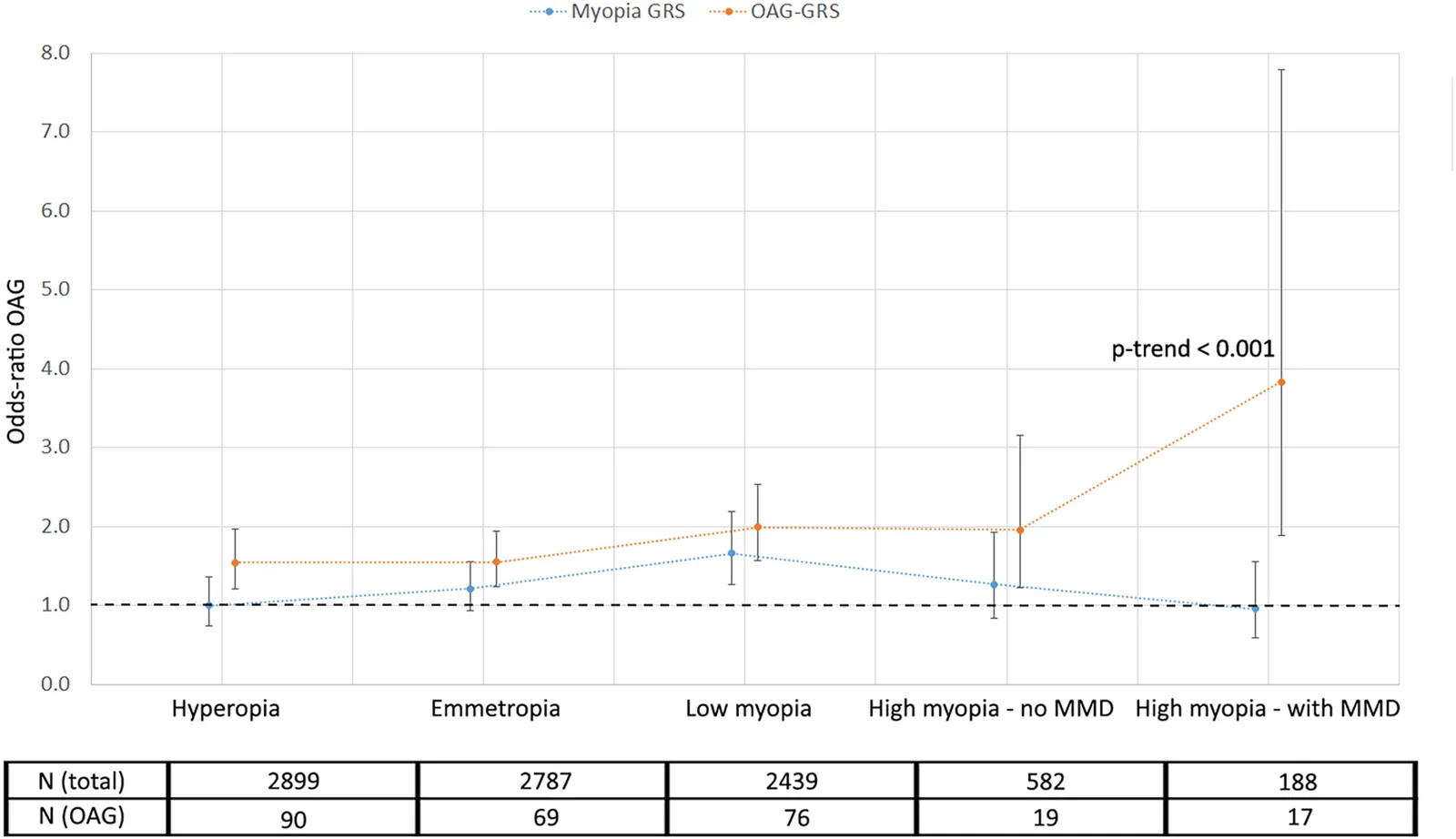
The association of a myopia GRS or OAG-GRS on OAG risk, stratified by stage of axial elongation and presence of MMD. Presented as the effect of a standard deviation increase in GRS. All associations were corrected for age and sex. GRS = genetic risk score, OAG = open-angle glaucoma, MMD = myopic macular degeneration.

The association of the OAG-GRS with OAG was equivalent within participants with hyperopia and emmetropia, with an OR (95% CI) of 1.54 (1.21, 1.97) and 1.55 (1.24, 1.94) per SD increase in GRS, respectively (Fig 2). This association was moderately stronger in both low myopia and high myopia without MMD, with an OR (95% CI) of 1.99 (1.57, 2.53) and 1.96 (1.22, 3.15) for each SD increase in GRS, respectively (Fig 2). However, the association of the OAG-GRS with OAG was substantially stronger specifically within those with high myopia with MMD, with an OR (95% CI) of 3.83 (1.89, 7.78) for each SD increase of the GRS. After stratification, the myopia GRS was only associated with OAG in the low myopes (Fig 2). The association of a myopia GRS with OAG was not statistically significant in hyperopia, emmetropia, or in high myopia (Fig 2).

### Pleiotropic Analysis Under a Composite Null Hypothesis

After clumping, 95 independent loci were identified with statistically significant evidence for pleiotropy between myopia and OAG (Fig S3, Table S7, available at www.ophthalmologyscience.org). A substantial majority of these loci (67 loci, 69.8%) congruently increased risk for both conditions, consistent with our GRS-based findings. Most lead SNPs were located either within the intron of a gene (47 SNPs, 49.4%), between genes (27 SNPs, 28.4%), or within the intron of a nonprotein coding RNA sequence (12 SNPs, 12.6%) (Fig S4, available at www.ophthalmologyscience.org). Genes annotated to the SNPs identified by PLACO were enriched in several molecular function pathways, the most significant of which was the pathway for structural constituents of chromatin (Fig S5, available at www.ophthalmologyscience.org). These genes and their associated pathways are the most likely to play key roles in the pathogenesis of both conditions and be future potential therapeutic targets.

## Discussion

In this large individual participant data meta-analysis, we found that a higher myopia GRS was associated with a higher prevalence of OAG, a higher IOP, and a larger VCDR. Conversely, a higher OAG-GRS was associated with longer axial length and more myopic spherical equivalent, suggestive of significant polygenic overlap, although the meta-analyses of the OAG-GRS showed substantial heterogeneity, which was only partially attenuated in the sensitivity analyses. High myopes with MMD and a high OAG-GRS were particularly likely to develop OAG. Finally, PLACO analysis identified 95 specific SNPs with statistically significant evidence for pleiotropy.

### Relation with Literature

Despite the well-known increased risk of OAG in myopia (especially high myopia), relatively little research has been done exploring a potentially shared genetic etiology. Two major studies investigated the relation between myopia and OAG using a 2-sample Mendelian randomization approach.^32^^,^^33^ However, although 2-sample Mendelian randomization uses GWAS data, it is designed to investigate the potential causal link between an outcome and an exposure, not a shared genetic architecture.^34^^,^^35^ In fact, 2-sample Mendelian randomization assumes there are no directly shared genetic factors (i.e., there is no horizontal pleiotropy), and the presence of horizontal pleiotropy is a violation of the study design.^33^^,^^34^ Given the previously reported widespread horizontal pleiotropy between complex traits and diseases, and our findings on the pleiotropy between myopia and OAG, it is unclear how to interpret 2-sample Mendelian randomization analyses between myopia and OAG. We would therefore interpret our own Mendelian randomization experiments as suggestive of substantial polygenic overlap, despite none of the SNPs being in direct linkage disequilibrium. Only 1 major study has investigated the potential pleiotropy between myopia and OAG more directly. Iglesias et al combined data from the Rotterdam Study and Australian & New Zealand Registry for Advanced Glaucoma Study, implementing an alternative GRS-based approach.^36^ They did not find an association of a myopia GRS with OAG (*P* = 0.81), IOP (*P* = 0.07), VCDR (*P* = 0.42), or optic cup area (*P* = 0.25), although they did find a significant association with retinal nerve fiber layer thickness (*P* = 7.7E-3).^12^ There are, however, some significant differences between methods and data used by Iglesias et al compared to our own approach. First, substantially less was known at the time about the genetic architecture of both diseases. Only 26 OAG genetic variants had been identified; this number has since increased more than 10-fold to 296 variants.^5^ Second, Iglesias et al only investigated the effect of a myopia GRS on OAG, not the effect of an OAG-GRS on myopia, possibly due to the very limited number of OAG variants available at that time. Third, because the cohorts used were a population-based study and an OAG clinical cohort, the number of included high myopes was very low. We instead utilized data of over 30 000 participants from 7 studies from the IGGC, including 1 high-myopia case–control study. Finally, statistical methods have been improved considerably in recent years. Iglesias et al were not able to apply any variant-specific statistical tests, whereas we leveraged PLACO to identify 95 specific loci with evidence for pleiotropy.

Genes annotated to the SNPs identified by PLACO were enriched in the pathway for structural constituents of chromatin (Figure S5). Among the most prominent loci identified in our PLACO analysis was rs10918274, an intronic variant in the *TMCO1* gene on chromosome 1 (Figure S6, available at www.ophthalmologyscience.org). This gene encodes a protein that is part of calcium channels in the endoplasmic reticulum and is one of the strongest and most consistently replicated genetic loci associated with OAG and IOP. Mutations in *TMCO1* have also been associated with craniofacial dysmorphism and skeletal abnormalities.^37^ There was another prominent locus on chromosome 10, rs1658436, an intronic variant in the *BICC1* gene (Figure S7, available at www.ophthalmologyscience.org). *BICC1* codes for an RNA-binding protein involved in the Wnt-signaling pathway, which is crucial for trabecular meshwork modulation, and disruption of this pathway has a high impact on IOP.^38^ This locus has also been associated with, among other traits, body height, hypertension, and renal dysplasia.^39^, ^40^, ^41^ Finally, the SNP with the strongest evidence for pleiotropy was rs34935520 on chromosome 14, an intergenic variant between the *SIX1* and *SIX6* genes, mutations in which have been associated with branchio-oto-renal syndrome, microphthalmia, and complex optic disc anomalies (Figure S8, available at www.ophthalmologyscience.org).^42^, ^43^, ^44^ Given the role of these SNPs in both (facial) dysplasias and ocular traits, and their strong evidence for pleiotropy in our PLACO analyses, a shared pleiotropic role in both OAG and myopia seems highly likely. More generally, changes in chromatin structure might occur in both conditions. Chromatin encompasses all DNA, and changes in its structure could therefore theoretically have a large range of effects. However, alterations in chromatin have been associated with changes in collagen expression.^45^, ^46^, ^47^ Collagen has been frequently linked to both myopia and OAG.^48^, ^49^, ^50^, ^51^ There is strong evidence for an association of variations in *COL15A1* and *COL18A1* with OAG, including a younger age of onset.^49^ Among others, variants in *COL1A1* and *COL2A1* have been associated with both high and low myopia.^50^ Mutations in collagen genes are also associated with syndromic Mendelian myopia, such as Stickler syndrome, in which high myopia is near universal and which can be caused by mutations in *COL2A1*, *COL11A1, COL9A2*, and *COL9A3*.^51^ Collagen changes, perhaps through altered expression patterns due to changes in chromatin structure, therefore constitute a likely shared pathway for both conditions.

### Strengths and Weaknesses

This study has several strengths. First, instead of using methods vulnerable to bias from (horizontal) pleiotropy, such as 2-sample Mendelian randomization or linkage disequilibrium score regression methods, we used a combination of one-sample Mendelian randomization and a single-variant pleiotropy test to demonstrate the substantial pleiotropic effects between both conditions. Second, for the one-sample Mendelian randomization tests, we utilized individual-level data from over 34 000 participants from 7 separate studies, which gave us enough data to detect bidirectional associations for almost all included exposure–outcome comparisons. Finally, we were able to include a large number of SNPs for our GRS analyses. The OAG-GRS validated well with consistent performance across all included studies. The myopia GRS also validated well in almost all studies, except for the Beijing Eye Study, which may be explained by the difference in ancestry of the Beijing Eye Study participants, or differences in environmental exposures (most notably near-work).

There are, however, also some limitations to consider. Firstly, the meta-analysis for the one-sample Mendelian randomization analyses showed significant heterogeneity. Heterogeneity was particularly high for the OAG-GRS analyses, possibly because of differences in OAG definition used in the underlying meta-GWAS, or because myopia is significantly more affected by environmental factors (i.e., the amount of near-work and outdoor time during childhood) than OAG, and genetic effects are therefore less consistent across studies from different areas across the world. For example, both Australian-based studies (Busselton Healthy Aging Study and the Raine Study) found mostly null findings in the OAG-GRS analyses. Despite being homogeneous with the rest of the cohorts in terms of having European ancestry, the most notable difference between these 2 studies and the others is that Australians spend relatively more time outdoors.^52^ A second limitation is that PLACO tests pleiotropy for 1 SNP at a time and therefore cannot detect pleiotropy between any particular SNP and a different SNP within linkage disequilibrium. PLACO therefore might be too conservative, although to our knowledge few methods have been developed for pleiotropy detection that explicitly consider linkage disequilibrium structure. Third, we did not include principal components as additional covariates in the regression analyses. Because the number of OAG and high-myopia cases in some of the smaller cohorts was not very high, the inclusion of principal components would go beyond the general rule-of-thumb of 10 degrees of freedom for every covariate. Although we did exclude any participants of the nonmajority ancestry, we cannot exclude some residual population stratification. Fourth, some of the meta-analyses, in particular those including the OAG-GRS, showed substantial heterogeneity, limiting the interpretability of the pooled estimates. Exclusion of the Beijing Eye Study and Dutch MYST did attenuate the heterogeneity somewhat, suggesting that our findings might not be directly generalizable to individuals of East Asian ancestry and/or high myopes. Finally, PLACO can also only detect the statistical association of a variant with 2 traits, referred to as “statistical pleiotropy,” and cannot distinguish between the various types of pleiotropy (i.e., direct biological pleiotropy, mediated pleiotropy, or pleiotropy due to strong linkage disequilibrium between causal variants in different genes).

## Conclusions

There is substantial evidence for a significant polygenic overlap between myopia and OAG, with a particularly high susceptibility to OAG in high myopes with MMD. We identified 95 specific loci with statistically significant evidence for pleiotropy, which warrants further research into the underlying biological mechanisms of these variants. Furthermore, an OAG-GRS may be helpful to clinically estimate OAG risk, in particular in challenging cases like those with high myopia and MMD.

## Supplementary Data

Supplementary Table S7

Collab Authors List
