# Supplementary material for: A Comprehensive Assessment of the Shared Genetic Architecture between Myopia and Open-Angle Glaucoma
Source: Ophthalmol Sci. 2026 Jul 22;6(10):101328. doi: 10.1016/j.xops.2026.101328 (PMC13553571; doi:10.1016/j.xops.2026.101328)
Supplement: Supplementary file 1 [file mmc1.pdf]

Supplementary Appendix

A comprehensive assessment of the shared genetic architecture between myopia and open-angle glaucoma

Contents

Supplementary Methods: Inclusion criteria, methods, and ethics statement of included studies.

Supplementary References

Supplementary Table S2: Overview of recruitment period, predominant ancestry, and study design of included studies.

Supplementary Table S3: Multivariable logistic regression of a standardized myopia GRS with high-myopia and the AUC for the GRS alone, with corresponding 95% confidence intervals.

Supplementary Table S4: Multivariable logistic regression of a standardized OAG GRS with OAG and the AUC for the GRS alone, with corresponding 95% confidence intervals.

Supplementary Table S6: The meta-analyzed association of a myopia GRS with OAG, IOP, and VCDR, and an OAG GRS with axial length, spherical equivalent, and high myopia. Excluding the Dutch Myopia Study and Beijing Eye Study.

Supplementary Table S7: Statistically significant SNPs (p = 5E-8) from the PLACO analysis.

Supplementary Figure S3: Manhattan plot of the PLACO p-values for pleiotropic association between myopia and OAG.

Supplementary Figure S4: Localization of PLACO significant SNPs with relation to genes or ncRNA regions

Supplementary Figure S5: Enrichment of genes identified by PLACO in molecular function pathways (GOMF gene-set MsigDB version c5).

Supplementary Figure S6: Regional association plots of significant pleiotropic locus rs10918274 (i.e. 1:165714416 in GRCh37), an intronic variant in the TMCO1 gene

Supplementary Figure S7: Regional association plots of significant pleiotropic locus rs1658436 (i.e. 10:60326910 in GRCh37), an intronic variant in the BICC1 gene

Supplementary Figure S8: Regional association plots of significant pleiotropic locus rs34935520 (i.e. 14:61091401 in GRCh37), an intergenic variant near the SIX1, SIX6, and C14orf39 genes

Supplementary Methods

Inclusion criteria, methods, and ethics statement of included studies.

EPIC-Norfolk

The European Prospective Investigation into Cancer (EPIC) is an international prospective cohort study, including more than 20 collaborating centers across 10 European countries, designed to investigate the relation of diet, nutrition, and lifestyle with the risk of cancer.[1] EPIC-Norfolk, one of the UK study sites, comprises a population-based cohort of 25,639 (response rate: 39.2%) participants aged 40–79 years at enrolment.[2] Participants were recruited from 35 participating general practices from across Norfolk and baseline assessments were conducted from 1993 to 1997. During the third health assessment (2004 to 2011), a comprehensive ophthalmic examination was performed in a subset of 8,623 participants aged 48–92 years (response rate: 60.4%).[3]

Glaucoma case ascertainment was determined from a detailed ophthalmic examination that included visual acuity, tonometry, assessment of the optic nerve head and peripapillary retinal nerve fiber layer (RNFL) with scanning laser ophthalmoscopy and polarimetry, and visual field assessment through automated perimetry.[3] Intraocular pressure (IOP) was measured with the AT-555 non-contact tonometer (Reichert, New York, United States) for the first 443 participants. For all subsequent participants (N = 5,721) IOP was measured with the Ocular Response Analyzer (Reichert, New York, United States). Vertical cup-to-disc ratio (VCDR) was measured using the Heidelberg Retina Tomograph (Heidelberg Engineering, Heidelberg, Germany). The IOLMaster V.4 (Carl Zeiss Meditech, Welwyn Garden City, United Kingdom) was

used for axial length measurements, and refractive error was measured with the Humphrey Auto-Refractor 500 (Humphrey Instruments, San Leandro, United States). Those participants with abnormal findings, based on predefined criteria, were referred for a definitive eye examination by a consultant ophthalmologist with a special interest in glaucoma. Full details of glaucoma case ascertainment in the EPIC-Norfolk Eye Study have been described previously.[3,4] Genotyping was performed using the Affymetrix (now Applied Biosystems) UK Biobank Axiom Array (Applied Biosystems, Santa Clara, United States). Genotypes were subsequently meta-imputed using both the Haplotype Reference Consortium (HRC) v1.0 panel and the UK10K plus 1000 Genomes phase 3 reference panel.[5]

All participants provided written informed consent and the study adhered to the tenets of the Declaration of Helsinki. The EPIC-Norfolk Study was approved by the Norwich Local Research Ethics Committee (05/Q0101/191) and the East Norfolk & Waveney NHS Research Governance Committee (2005EC07L).

Gutenberg Health Study (GHS)

The Gutenberg Health Study (GHS) is a population-based, prospective single-center cohort study with a total sample size of 15,010 individuals conducted in the Rhine-Main region in midwestern Germany. The study design has been published elsewhere in detail.[6,7] Briefly, individuals between the ages of 35 and 74 years were randomly selected from local governmental registries with a sampling procedure that was stratified for sex, residential area, and age. Participant recruitment ran from 2007 to 2012. The baseline investigation involved a

standardized clinical examination performed by trained and certified medical technical assistants according to a uniform written protocol.

IOP was measured using the Nidek NT-188 2000 non-contact tonometer (Nidek Co, Gamagori, Japan). Assessment of VCDR was primarily performed on 30° optic nerve head-centered color fundus photographs which were acquired with the Visucam PRO NM non-mydriatic camera (Carl Zeiss, Oberkochen, Germany), and if image quality did not allow measurement in 30° images, in 45° images. Minimal rim-to-disc ratio was measured between 11 and 1 o'clock respective to 5 and 7 o'clock. Optic disc area calculation was conducted based on horizontal and vertical disc diameter and assuming an ellipse shape. Optical magnification was incorporated using Littmann correction and data on refraction and keratometry. Autorefraction and corrected visual acuity were measured using a Humphrey Automated Refractor / Keratometer (HARK) 599 (Carl Zeiss, Oberkochen, Germany). Axial length was measured using the Lenstar LS900 (Haag-Streit, Koeniz, Switzerland). Visual field was tested using the Frequency Doubling Technology Humphrey Matrix Perimeter (Carl Zeiss, Oberkochen, Germany). The presence of a visual field defect was determined if we observed at least 2 abnormal fields (P<1%). Glaucoma cases were classified according to ISGEO criteria categories 1 and 2.[8] Only definite cases were included in this analysis. The percentiles for the ISGEO criteria were calculated using a hypernormal subset (only subjects without visual field defects) as reference. We developed quantile regression formulas to calculate individual cut-off values for VCDR with respect to disc area with an upper limit of 0.8. Slit lamp examination of the anterior segment was conducted by an ophthalmologist and, in case of a narrow anterior chamber angle, this was documented. No gonioscopy was conducted. Pseudoexfoliation and pigment

dispersion was assessed through slit lamp examination. Glaucoma was categorized as open-angle glaucoma (OAG) if no narrow anterior chamber angle was documented. Genomic DNA extraction was obtained from two buffy-coated EDTA blood samples from each participant. DNA samples were genotyped using the Illumina Omni 2.5 array (Illumina, San Diego, United States) and imputed with the 1000 Genomes phase 3 reference panel.

Written informed consent was obtained from all individual participants included in the study and all procedures performed in the GHS were approved by the ethics committee of the Medical Chamber of the State of Rhineland-Palatinate and the local data safety commissioners (837.020.07(5555)). The study design is in accordance with the revised Helsinki protocol and principles outlined in recommendations for Good Clinical and Epidemiological Practice.

Rotterdam Study (RS)

The Rotterdam Study is a prospective population-based cohort study of people living in Ommoord, a district of the city of Rotterdam.[9] The RS consists of four cohorts, of which the first three (RS-I, RS-II, and RS-III) were used for this study (the fourth is still ongoing). Inclusion of these three cohorts started in 1991, 2000, and 2006, respectively. Participants were examined every 4 to 5 years following a standardized protocol, which included visual field testing at baseline and during follow-up for the presence of OAG. Participants underwent an extensive physical examination at the research center as well as a home interview including questionnaires on lifestyle factors and medical history. Details have been published previously.[10]

In short, the IOP was measured using Goldmann applanation tonometry (Haag-Streit, Koeniz, Switzerland), except for the last visit of RS-I (RS-I-7), which was measured using a Topcon TRK-2P non-contact tonometer (Topcon, Tokyo, Japan). Data on usage of IOP-lowering medications (including anatomic therapeutic chemical codes) was obtained from 7 fully automated pharmacies using a centralized computer network in the study district from January 1, 1991, onward. VCDR measurements were extracted from simultaneous stereo color photographs of the optic nerve head (Topcon ImageNet System; Topcon, Tokyo, Japan), or the Heidelberg Retina Tomograph (HRT; Heidelberg Engineering, Dossenheim, Germany) was used to calculate the linear cup-to-disc ratio, which was used as an equivalent outcome for the VCDR analyses (N with linear CDR = 3,615, 33.2% of the total cohort).[11] Refractive error was measured using the Topcon TRK-2P refractometer (Topcon, Tokyo, Japan), and axial length was measured using the Lenstar LS900 (Haag Streit, Koeniz, Switserland). All participants underwent visual field testing. Visual field loss had to be reproducible on 2 screening visual field tests (Humphrey Visual Field Analyzer [HFA] II 740 or Frequency Doubling Technology Humphrey Matrix Perimeter; Carl Zeiss, Oberkochen, Germany) and a subsequent HFA 24-2 full threshold or SITA standard test. Visual field loss was considered glaucomatous if no other cause could be identified, and no homonymous defects or recognizable patterns like rim artifacts were observed. Hospital records were retrieved for all participants with visual field loss. Participants with other possible causes of visual field loss, signs of anterior chamber angle-closure, secondary glaucoma, pseudoexfoliation, or pigment dispersion syndrome were excluded. OAG was defined as glaucomatous visual field loss in at least one eye, independent of IOP. DNA extraction was performed using whole blood samples. Genotyping was performed using both

the Infinium II HumanHap550(-Duo) (RS-I and RS-II) and 610-Quad Genotyping BeadChip (RS-I and RS-III; Illumina, San Diego, CA, USA). Imputation of markers was performed using the TOPMed reference panel.

The Medical Ethics Committee of Erasmus MC (registration number MEC 02.1015) and the Dutch Ministry of Health, Welfare, and Sport (Population Screening Act WBO, license number 1071272-159521-PG) approved the use of the individual-level data in the Rotterdam Study participants. Personal registration data collection is filed with the Erasmus MC Data Protection Officer under registration number EMC1712001. The Rotterdam Study is entered into the Netherlands National Trial Register (NTR; www.onderzoekmetmensen.nl) and the World Health Organization (WHO) International Clinical Trials Registry Platform (ICTRP; www.who.int/ictrp/network/primary/en/) under shared catalog number NL6645/NTR6831. All participants provided written informed consent following the Declaration of Helsinki.

Busselton Healthy Aging Study (BHAS)

The BHAS is a community-based cohort study based in the City of Busselton, Western Australia.[12] In 2010, non-institutionalized residents born between 1946 and 1964 who were registered on the electoral roll for the Local Government Area of Busselton were randomly selected to participate in the BHAS. Phase 1 of the study was conducted in 2010–2015, during which participants underwent a myriad of health examinations. IOP was measured with the iCare TAOLi rebound tonometer (iCare Finland Oy, Helsinki, Finland). Fundus photographs were acquired with the Canon CR-1 fundus camera (Canon, Tokyo, Japan). The VCDR of

participants were then manually graded by an experienced observer for a subset of these participants. Autorefraction and keratometry measurements were taken using a handheld Auto Refractor / Keratometer ARK-30 (Nidek, Gamagori, Japan). Axial length data was not collected. OAG was based on self-reports of diagnosis or medication usage, corroborated with data linkage information from electronic patient health records.

Whole blood samples were collected from participants, from which DNA was extracted and genotyped using an Illumina 660W Quad Array Bead Chip (Illumina, San Diego, United States). Generated data with a SNP call rate < 0.95 and Hardy-Weinberg Equilibrium p< 10-6 were removed. Imputation was conducted against the TOPMed reference panel, and those with imputation quality r>0.3 were retained in the analysis.

This study was planned and conducted in accordance with the principles of the Declaration of Helsinki, and was approved by the University of Western Australia Human Research Ethics Committee (Number 2021/ET000260). Written informed consent was obtained from all participants.

The Raine Study Generation 2

In 1989–1991, 2,900 pregnant women were recruited from obstetric clinics in Perth, Western Australia.[13] The study explored the effect of frequent ultrasound imaging during pregnancy on birth outcomes and formed a cohort for studying the effects of early life events on later health outcomes. The women were randomly assigned to an “intensive imaging” group or the control group.[14] Children born to these women (n= 2,868) formed the original study cohort (Gen2) and have been undergoing a series of various health and medical examinations from birth. With the

enrolment of their parents (Gen1), grandparents (Gen0), and children (Gen3), the Raine Study has become one of the longest running multigenerational cohort studies in the world.

The Gen2-20 year follow-up was conducted from March 2010 to February 2012, when 1,344 participants (46.9% of the original cohort of 2,868) had their first eye examination as part of the study.[15] Refractive error was measured after cycloplegia with the Nidek ARK-510A (Nidek Co, Gamagori, Japan). Axial length was measured with the IOLMaster V.5 (Carl Zeiss, Oberkochen, Germany). Tonometry was conducted using an iCare TA01i rebound tonometer (iCare Finland, Vantaa, Finland). The VCDR was determined by optical coherence tomography (OCT) imaging with a Spectralis HRA+ OCT (Heidelberg Engineering, Heidelberg, Germany). Finally, participants were genotyped using the Infinium HD Human 660W-Quad BeadChip Array (Illumina, San Diego, United States). Genotype data was subsequently imputed on the TOPMed reference panel.

The study was conducted in accordance with the tenets of the Declaration of Helsinki, and ethical approval for each follow-up was obtained from the University of Western Australia's Human Research Ethics Committee. All participants provided written informed consent before participating.

Beijing Eye Study (BES)

The BES is a cross-sectional population-based study in Beijing, China. The only eligibility criterion for inclusion in the study was an age of over 50 years. In 2011, it had a total population of 4,403 individuals aged 50 years or older. Information on demographic variables, socioeconomic background, and known major systemic diseases were collected.

All subjects underwent comprehensive ophthalmological examination. Visual acuity, best corrected visual acuity, and IOP were recorded before pupil dilatation. Refractive error was measured with the Nidek Auto Refractometer AR-610 (Nidek Co, Gamagori, Japan), and IOP with Goldmann applanation tonometry (Haag-Streit, Koeniz, Switzerland). Axial length was measured with the Lenstar LS 900 (Haag-Streit, Koeniz, Switzerland). VCDR was determined by manual annotation of the cup and disc borders on fundus photographs acquired with the Type CR6-45NM Canon camera (Canon, Tokyo, Japan). Slit lamp examination, indirect ophthalmoscope examination, anterior segment, and fundus examination were performed. The presence of glaucoma was determined by qualitative assessment of the optic disc by a single examiner under supervision of two glaucoma experts.[14] Further details have been described previously.[16] Genotyping was performed in two batches. The first batch was genotyped on Illumina 610k array (N = 538) and the second with the Infinium OmniExpress array (N = 476)(Illumina, San Diego, United States). Batches were imputed as a combined dataset using the 1000 Genomes Asia Project reference panel.

The Medical Ethics Committee of the Beijing Tongren Hospital approved the study protocol and all participants gave informed written consent, according to the Declaration of Helsinki.

Dutch Myopia Study (MYST)

The Dutch MYopia STudy (MYST), is a high myopia case control study which was executed in 2010-2020.[17] Participants were recruited for MYST via public media, eye care providers and announcements on the study website and Erasmus Medical Center website. Persons with

syndromic high myopia were excluded. Inclusion criteria for high-myopia cases were a mean spherical equivalent ≤ -6.0 diopters or axial length ≥ 26.0 mm in case of bilateral pseudophakia, aphakia or refractive surgical procedures without data on refractive error before surgery. High myopia cases and controls were recalculated according to the definition used in this meta-analysis (i.e. an axial length of ≥ 26.0 mm, or if axial length was not available a spherical equivalent of ≤ -6.0 D, excluding pseudophakic participants and participants with a history of refractive-surgery).

Participation in the MYST study included extensive ophthalmological examination following a standardized protocol. This included refractive error measurements with the Topcon RM-A2000 Auto-Refractor (Topcon, Tokyo, Japan), and axial length measurements with Lenstar LS900 (Haag-Streit, Koeniz, Switzerland), or with the A-scan function of PacScan 300 AP (Sonomed Escalon, Westbury, United States) for participants with an axial length of 30 mm or greater. IOP was measured with Goldmann applanation tonometry (Haag-Streit, Koeniz, Switzerland). After pharmacologic mydriasis with phenylephrine and tropicamide, we performed retinal imaging including stereoscopic 35° digital color fundus photography of the macula and optic disc using the Topcon TRC-50EX (Topcon, Tokyo, Japan) with a Sony DXC-950P digital camera, 0.44 megapixel (Sony Corporation, Tokyo, Japan). OCT images were acquired with the Topcon 1000 mkII 3D-OCT and Topcon 2000 3D-OCT (Topcon, Tokyo, Japan). The retinal images were graded using information from all modalities (fundus photography, OCT, and autofluorescence). The color fundus photographs were graded according to META-PM criteria.[17] Genotyping was performed on the Illumina Omni Express array and imputed on the 1000 Genomes phase 3 reference panel.

This study adhered to the tenets of the Declaration of Helsinki and was approved by the local ethics committees of Erasmus Medical Center, Rotterdam, the Netherlands (MEC 2009-248). Written informed consent was obtained from all study participants.

Supplementary References

1. Riboli E, Kaaks R. The EPIC Project: rationale and study design. European Prospective Investigation into Cancer and Nutrition. Int J Epidemiol 1997;26:S6-14.

2. Day N, Oakes S, Luben R, et al. EPIC-Norfolk: study design and characteristics of the cohort. European Prospective Investigation of Cancer. Br J Cancer 1999;80:95–103.

3. Khawaja AP, Chan MPY, Hayat S, et al. The EPIC-Norfolk Eye Study: rationale, methods and a cross-sectional analysis of visual impairment in a population-based cohort. BMJ Open 2013;3:e002684.

4. Chan MPY, Broadway DC, Khawaja AP, et al. Glaucoma and intraocular pressure in EPICNorfolk Eye Study: cross sectional study. BMJ 2017;358:j3889.

5. Yu K, Das S, LeFaive J, Kwong A, Pleiness J, Forer L, Schönherr S, Fuchsberger C, Smith AV, Abecasis GR. Meta-imputation: An efficient method to combine genotype data after imputation with multiple reference panels. Am J Hum Genet. 2022 Jun 2;109(6):1007-1015. doi: 10.1016/j.ajhg.2022.04.002. Epub 2022 May 3. PMID: 35508176; PMCID: PMC9247833.

6. Wild PS, Zeller T, Beutel M, et al. The Gutenberg Health Study. Bundesgesundheitsblatt Gesundheitsforschung Gesundheitsschutz 2012;55:824–9. 17.

7. Höhn R, Kottler U, Peto T, et al. The ophthalmic branch of the Gutenberg Health Study: study design, cohort profile and self-reported diseases. PLoS One 2015;10:e0120476.

8. Foster PJ, Buhrmann R, Quigley HA, Johnson GJ. The definition and classification of glaucoma in prevalence surveys. Br J Ophthalmol. 2002 Feb;86(2):238-42. doi: 10.1136/bjo.86.2.238. PMID: 11815354; PMCID: PMC1771026.

9. Ikram MA, Brusselle G, Ghanbari M, et al. Objectives, design and main findings until 2020 from the Rotterdam Study. Eur J Epidemiol. 2020; 35(5): 483–517.

10. de Vries VA, Hanyuda A, Vergroesen JE, et al. The clinical utility of a glaucoma polygenic risk score in four population-based European-ancestry cohorts. Ophthalmology. 2025; 132(2): 228–237.

11. Ramdas WD, Wolfs RC, Hofman A, de Jong PT, Vingerling JR, Jansonius NM. Heidelberg retina tomograph (HRT3) in population-based epidemiology: normative values and criteria for glaucomatous optic neuropathy. Ophthalmic Epidemiol. 2011; 18(5): 198–210. Erratum in: Ophthalmic Epidemiol. 2012;19(2):116.

12. James A, Hunter M, Straker L, et al; Busselton Healthy Ageing Study Investigator Group. Rationale, design and methods for a community-based study of clustering and cumulative effects of chronic disease processes and their effects on ageing: the Busselton healthy ageing study. BMC Public Health. 2013;13:936. doi:10.1186/1471-2458-13-936

13. McKnight CM, Newnham JP, Stanley FJ, Mountain JA, Landau LI, Beilin LJ, et al. Birth of a cohort–the first 20 years of the Raine study. Med J Aust. (2012) 197:608–10. 10.5694/mja12.10698

14. Newnham JP, Evans SF, Michael CA, Stanley FJ, Landau LI. Effects of frequent ultrasound during pregnancy: a randomised controlled trial. Lancet. (1993) 342:887–91. 10.1016/0140-6736(93)91944-H

15. Yazar S, Forward H, McKnight CM, Tan A, Soloshenko A, Oates SK, Ang W, Sherwin JC, Wood D, Mountain JA, Pennell CE, Hewitt AW, Mackey DA. Raine eye health study: design, methodology and baseline prevalence of ophthalmic disease in a birth-cohort study of young

adults. Ophthalmic Genet. 2013 Dec;34(4):199-208. doi: 10.3109/13816810.2012.755632. Epub 2013 Jan 10. PMID: 23301674.

16. Shao L, Xu L, Wei WB, Chen CX, Du KF, Li XP, Yang M, Wang YX, You QS, Jonas JB. Visual acuity and subfoveal choroidal thickness: the Beijing Eye Study. Am J Ophthalmol. 2014;158(4):702–709.e701. doi: 10.1016/j.ajo.2014.05.023.

17. Xu L, Wang Y, Wang S, Wang Y, Jonas JB. High myopia and glaucoma susceptibility the Beijing Eye Study. Ophthalmology. 2007 Feb;114(2):216-20. doi: 10.1016/j.ophtha.2006.06.050. Epub 2006 Nov 21. PMID: 17123613.

18. Haarman AEG, Tedja MS, Brussee C, Enthoven CA, van Rijn GA, Vingerling JR, Keunen JEE, Boon CJF, Geerards AJM, Luyten GPM, Verhoeven VJM, Klaver CCW. Prevalence of Myopic Macular Features in Dutch Individuals of European Ancestry With High Myopia. JAMA Ophthalmol. 2022 Feb 1;140(2):115-123. doi: 10.1001/jamaophthalmol.2021.5346. PMID: 34913968; PMCID: PMC8678902.

19. Ohno-Matsui K, Kawasaki R, Jonas JB, et al. ; META-analysis for Pathologic Myopia (META-PM) Study Group . International photographic classification and grading system for myopic maculopathy. Am J Ophthalmol. 2015;159(5):877-83.e7. doi: 10.1016/j.ajo.2015.01.022
