# Supplementary material for: A Comprehensive Assessment of the Shared Genetic Architecture between Myopia and Open-Angle Glaucoma
Source: Ophthalmol Sci. 2026 Jul 22;6(10):101328. doi: 10.1016/j.xops.2026.101328 (PMC13553571; doi:10.1016/j.xops.2026.101328)
Supplement: Supplementary file 2 [file mmc10.pdf]

Table S6. The meta-analyzed logistic and linear regression of a myopia GRS with OAG, IOP, and VCDR, and an OAG GRS with high myopia, axial length, and spherical equivalent. Excluding the Dutch Myopia Study and Beijing Eye Study.

**Myopia GRS**	**Total N**	**Continuous**		**Q1**	**Q2**	**Q3**	**Q4**
OAG, OR (95% CI)	29,268	1.17 (1.08, 1.27)*		1.0	1.26 (1.00, 1.59)*	1.19 (0.96, 1.46)	1.36 (1.11, 1.66)*
OAG, matched†, OR (95% CI)	2,364	1.17 (0.91, 1.50)		1.0	1.46 (0.87, 2.43)	1.34 (0.99, 1.81)	1.43 (0.67, 3.07)
IOP, beta (95% CI)	32,332	0.04 (0.00, 0.08)		0.0	0.11 (0.02, 0.21)*	0.05 (-0.05, 0.14)	0.12 (0.02, 0.22)*
IOP, adjusted‡, beta (95% CI)	32,332	0.12 (-0.03, 0.28)		0.0	0.10 (0.00, 0.20)*	0.06 (-0.04, 0.16)	0.14 (0.04, 0.25)*
VCDR, beta (95% CI)	27,214	0.005 (0.003, 0.006)*		0.0	0.003 (-0.002, 0.008)	0.006 (0.001, 0.011)*	0.005 (0.003, 0.006)*
**OAG GRS**	**Total N**	**Continuous**		**Q1**	**Q2**	**Q3**	**Q4**
High myopia, OR (95% CI)	32,243	1.02 (0.91, 1.15)		1.0	1.10 (0.94, 1.28)	1.13 (0.86, 1.45)	1.07 (0.76, 1.50)
Axial length, beta (95% CI)	25,342	0.04 (0.01, 0.09)*		0.0	0.02 (-0.03, 0.07)	0.06 (-0.01, 0.12)	0.12 (0.01, 0.23)*
Spherical equivalent, beta (95% CI)	31,118	-0.05 (-0.10, 0.00)		0.0	-0.01 (-0.08, 0.06)	-0.06 (-0.12, 0.01)	-0.07 (-0.14, -0.01)*

Presented as the effect of one standard deviation increase in GRS (continuous) and stratified into quartiles, with the lowest quartile as the reference category. All associations were corrected for age and sex.

* = $p < 0.05$

† = Matched on axial length with a 1:3 case:control ratio

‡ = adjusted for IOP-lowering medications (IOP divided by 0.7) and IOP-lowering surgery (IOP set to 30 mmHg if IOP was below 30 mmHg prior to adjustment)

GRS = genetic risk score, OAG = open-angle glaucoma, IOP = intraocular pressure, VCDR = vertical cup-to-disc ratio, Q = quartile, OR = odds-ratio
