# Supplementary material for: A Comprehensive Assessment of the Shared Genetic Architecture between Myopia and Open-Angle Glaucoma
Source: Ophthalmol Sci. 2026 Jul 22;6(10):101328. doi: 10.1016/j.xops.2026.101328 (PMC13553571; doi:10.1016/j.xops.2026.101328)
Supplement: Supplementary file 3 [file mmc11.pdf]

Table S7. Statistically significant SNPs (p = 5E-8) from the PLACO analysis.

Chromosome	Location*	Effect allele	Reference allele	P-value OAG GWAS	P-value myopia GWAS	P-value PLACO
1	42309581	C	G	1.81E-04	1.34E-15	2.91E-11
1	48114417	T	G	1.63E-05	1.52E-07	4.85E-08
1	54172944	A	G	9.11E-10	3.60E-10	8.09E-13
1	61335938	T	C	2.14E-06	6.47E-15	1.63E-13
1	92068967	A	G	1.11E-09	1.55E-05	4.02E-09
1	113233698	T	C	8.11E-10	1.40E-04	4.48E-09
1	165714416	T	C	1.66E-92	5.10E-08	1.09E-32
1	219778675	T	G	1.07E-05	3.56E-29	7.01E-18
2	12867992	T	C	4.91E-11	2.55E-04	1.96E-08
2	45143739	C	G	3.11E-05	1.83E-14	5.66E-12
2	55933014	T	C	1.37E-18	6.97E-05	1.41E-11
2	71534161	C	G	5.20E-09	3.89E-06	2.32E-09
2	178853378	A	G	7.29E-03	8.05E-53	2.13E-12
2	233259772	A	G	0.03	1.61E-22	8.80E-09
3	8191247	T	C	0.13	8.23E-35	2.37E-08
3	24231030	C	G	5.32E-03	1.39E-21	7.06E-09
3	49980596	T	C	6.32E-04	3.06E-11	6.76E-09
3	66861887	A	G	1.12E-07	7.32E-06	2.01E-08
3	85168434	T	C	2.88E-16	9.10E-06	4.35E-12
3	85264266	A	G	4.39E-08	1.89E-05	2.69E-08
3	85370873	T	C	2.73E-07	4.76E-06	2.43E-08
3	85644911	T	C	2.17E-06	8.99E-14	7.31E-12
3	98972835	A	G	1.54E-07	6.01E-12	4.08E-12
3	141061438	A	G	2.47E-03	1.42E-21	1.40E-09
3	171820211	T	G	2.61E-17	7.28E-11	1.55E-19
4	7898044	T	C	1.04E-50	2.93E-04	1.54E-16
4	81944558	A	G	1.49E-03	6.18E-24	1.75E-10
4	89752276	A	G	4.22E-08	6.59E-22	7.13E-17
5	71700591	A	G	4.22E-07	7.11E-10	1.16E-10
6	634835	A	T	1.09E-16	1.01E-03	3.09E-09
6	1776194	T	C	2.42E-11	3.84E-06	1.77E-10
6	2433054	T	C	2.04E-06	2.25E-13	1.24E-11
6	29782633	T	C	1.55E-06	9.08E-09	2.23E-10
6	31641139	A	G	1.11E-07	1.53E-09	6.53E-12
6	32582577	A	C	6.14E-05	2.92E-09	3.50E-09
6	36570366	T	C	5.72E-17	5.56E-04	4.42E-11
6	51438609	T	C	2.78E-26	1.69E-07	9.46E-20
6	73598305	T	C	0.01	6.56E-88	2.23E-13
6	129817167	T	G	5.38E-03	2.14E-63	8.53E-14

7	39057109	A	G	6.47E-09	6.55E-08	1.04E-10
7	116153025	A	C	2.02E-25	3.77E-07	6.93E-17
8	40707884	A	C	3.28E-03	1.76E-29	1.22E-10
8	60178580	C	G	0.05	2.76e-106	8.16E-11
8	78964111	C	G	3.69E-05	6.65E-12	9.56E-10
8	108294080	T	C	1.25E-15	2.97E-04	8.73E-10
9	76454263	T	C	1.87E-05	8.71E-09	1.87E-09
9	107692229	A	G	7.05E-72	3.00E-03	1.85E-15
9	129386860	T	C	3.37E-38	3.10E-04	1.56E-14
9	136131461	A	G	1.01E-10	4.77E-07	4.75E-11
10	60326910	A	G	6.03E-22	1.79E-29	1.37E-33
10	79159158	C	G	0.02	1.42E-36	4.97E-09
10	85937758	T	C	6.15E-04	5.88E-27	7.07E-12
10	90024599	A	G	6.41E-06	1.72E-19	2.48E-13
10	94946268	T	G	5.77E-11	2.57E-15	1.03E-16
10	96039597	C	G	4.93E-14	2.98E-05	7.86E-12
10	114791490	T	C	4.05E-05	6.23E-25	1.05E-13
11	28643299	T	C	2.90E-04	2.90E-12	9.65E-09
11	40156319	T	C	0.05	3.97E-42	3.07E-08
11	47469439	A	G	4.36E-14	2.61E-10	1.34E-15
11	47902883	A	G	5.93E-06	2.42E-10	7.64E-10
11	86307190	A	G	2.63E-11	9.96E-06	4.31E-10
11	120207405	A	G	1.68E-19	3.50E-03	2.61E-10
12	6954864	A	G	1.86E-05	7.75E-47	7.57E-19
12	28203245	A	T	1.12E-08	1.65E-04	2.04E-08
13	28966067	A	G	2.72E-05	5.50E-14	7.74E-12
13	50122681	A	G	4.51E-03	1.52E-26	6.46E-10
13	85554743	T	G	7.57E-03	1.54E-14	2.89E-08
13	100794886	T	C	0.03	1.59E-25	2.39E-09
14	33259064	A	G	0.01	9.95E-17	2.29E-08
14	42294993	A	G	3.44E-03	3.44E-22	1.99E-09
14	54421052	T	G	7.99E-03	7.24E-34	3.39E-10
14	61091401	A	G	3.07E-61	1.05E-34	1.83E-61
14	74929721	T	C	1.11E-07	1.32E-16	2.36E-14
15	35005886	A	T	2.89E-04	1.35e-171	7.22E-27
15	50999698	A	G	2.24E-04	4.97E-10	4.25E-08
15	63574641	A	G	2.30E-03	9.61E-24	4.20E-10
15	66989515	T	C	1.54E-10	4.14E-11	6.57E-14
15	74229065	A	G	2.13E-09	4.56E-15	2.23E-15
16	7456936	A	T	3.30E-03	5.92E-44	1.22E-12
17	11429715	A	T	4.10E-05	1.84E-43	1.72E-17
17	14133350	T	G	4.89E-03	8.27E-17	4.99E-08

17	43541656	T	C	2.75E-07	3.52E-09	2.65E-11
17	44025888	A	C	8.80E-10	9.21E-09	5.71E-13
17	47280915	T	C	2.08E-05	4.16E-36	1.06E-16
17	55564743	A	C	6.47E-07	3.57E-06	3.42E-08
17	58878665	T	G	1.08E-08	7.93E-12	3.04E-14
17	68718734	T	C	1.20E-03	2.18E-17	2.63E-09
17	79583371	A	C	7.07E-03	4.18E-30	6.43E-10
18	42803331	T	C	1.17E-03	6.08E-14	2.11E-08
20	6760344	A	G	8.12E-05	3.13E-26	1.90E-15
21	16327357	A	C	3.16E-04	1.59E-12	8.67E-09
21	39908003	T	C	4.49E-05	7.33E-09	4.24E-09
22	38121152	A	C	2.63E-12	2.15E-03	1.60E-08
22	41640850	T	C	3.07E-04	3.41E-10	7.37E-09
22	46376384	A	G	1.05E-05	2.82E-26	6.32E-15

* Location in Genome Reference Consortium human build 37

PLACO = pleiotropic analysis under composite null hypothesis, SNP = single nucleotide polymorphism, OAG = open-angle glaucoma, GWAS = genome-wide association study
