# Supplementary material for: A Comprehensive Assessment of the Shared Genetic Architecture between Myopia and Open-Angle Glaucoma
Source: Ophthalmol Sci. 2026 Jul 22;6(10):101328. doi: 10.1016/j.xops.2026.101328 (PMC13553571; doi:10.1016/j.xops.2026.101328)
Supplement: Supplementary file 4 [file mmc2.pdf]

Figure S3. Manhattan plot of the PLACO p-values for pleiotropic association between myopia and OAG.

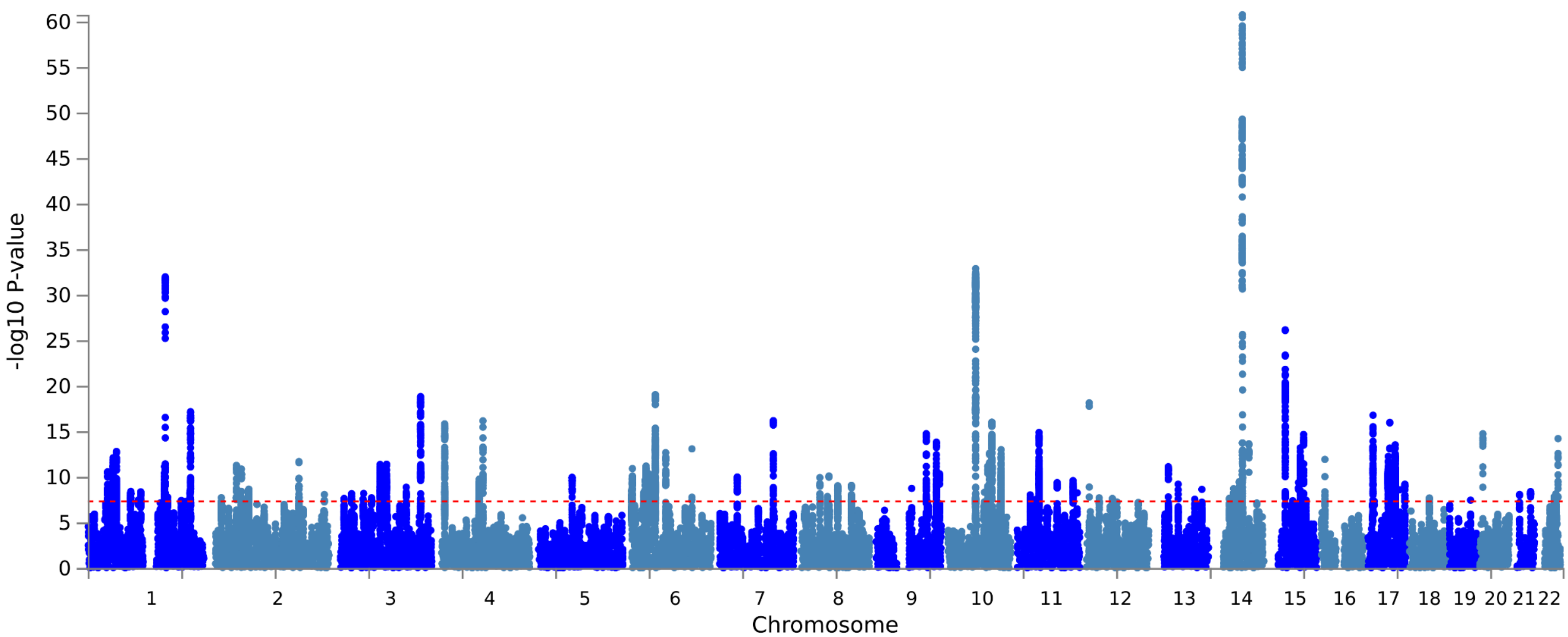

Red dotted line indicates genome-wide significance (p <5E-8).
PLACO = pleiotropic analysis under composite null hypothesis, OAG = open-angle glaucoma
