# Supplementary material for: A Comprehensive Assessment of the Shared Genetic Architecture between Myopia and Open-Angle Glaucoma
Source: Ophthalmol Sci. 2026 Jul 22;6(10):101328. doi: 10.1016/j.xops.2026.101328 (PMC13553571; doi:10.1016/j.xops.2026.101328)
Supplement: Supplementary file 5 [file mmc3.pdf]

Figure S4. Localization of PLACO significant SNPs with relation to genes or ncRNA regions

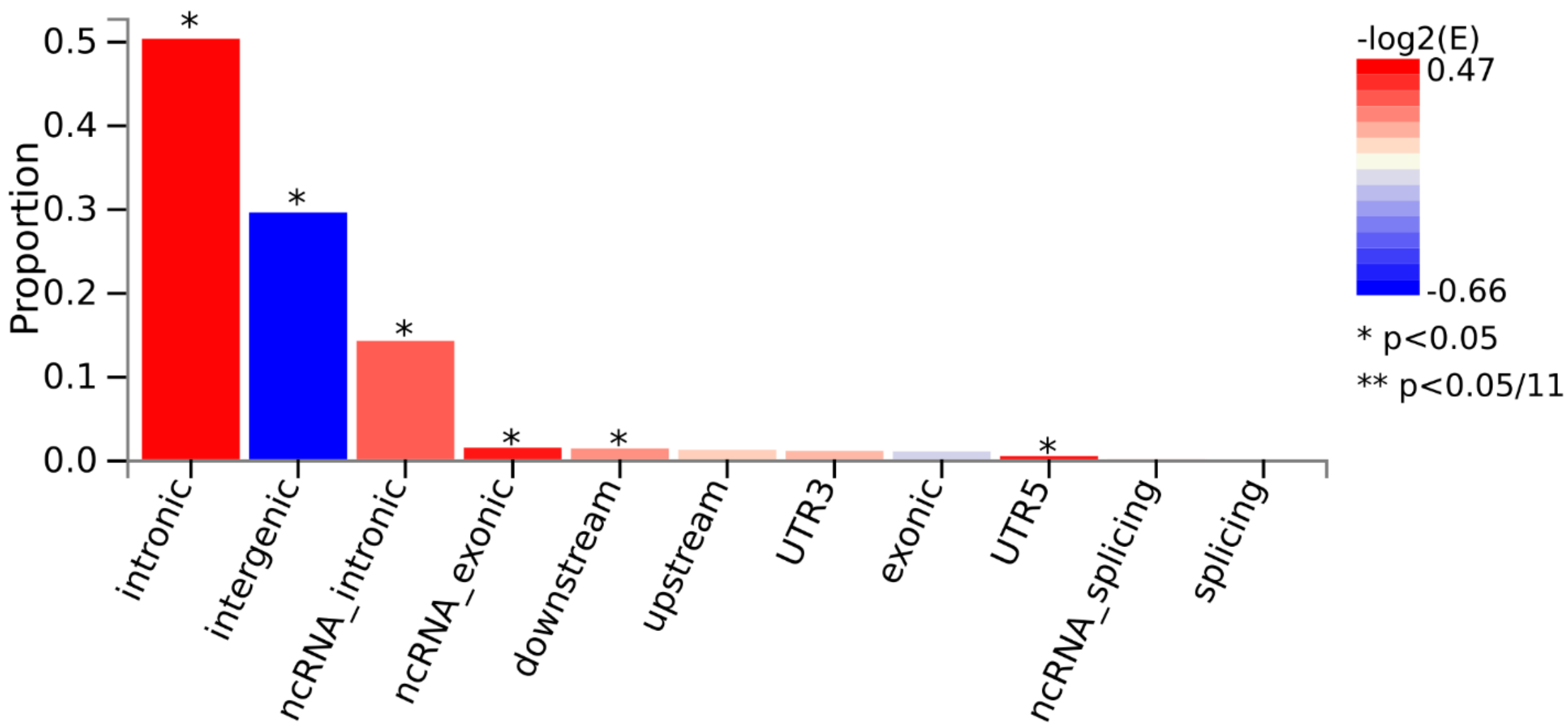

PLACO = pleiotropic analysis under composite null hypothesis, SNP = single nucleotide polymorphism, ncRNA = non-coding RNA
