# Supplementary material for: A Comprehensive Assessment of the Shared Genetic Architecture between Myopia and Open-Angle Glaucoma
Source: Ophthalmol Sci. 2026 Jul 22;6(10):101328. doi: 10.1016/j.xops.2026.101328 (PMC13553571; doi:10.1016/j.xops.2026.101328)
Supplement: Supplementary file 6 [file mmc4.pdf]

**Figure S5.** Enrichment of genes identified by PLACO in molecular function pathways (GOMF gene-set MsigDB version c5).

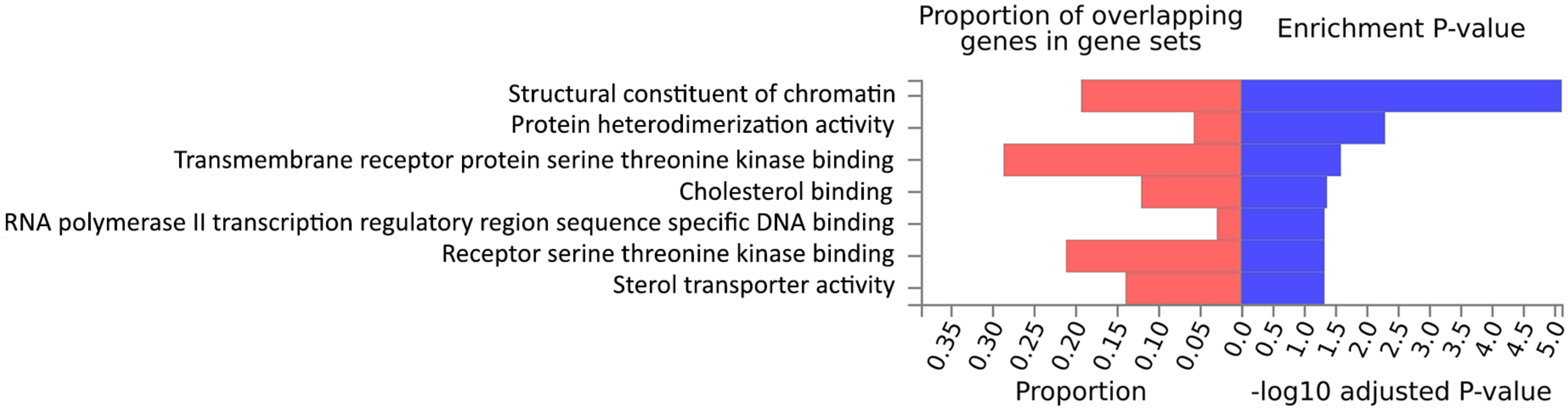


PLACO = pleiotropic analysis under composite null hypothesis, GOMF = gene ontology molecular functions
