# Supplementary material for: A Comprehensive Assessment of the Shared Genetic Architecture between Myopia and Open-Angle Glaucoma
Source: Ophthalmol Sci. 2026 Jul 22;6(10):101328. doi: 10.1016/j.xops.2026.101328 (PMC13553571; doi:10.1016/j.xops.2026.101328)
Supplement: Supplementary file 8 [file mmc6.pdf]

Figure S7. Regional association plots of significant pleiotropic locus rs1658436 (i.e. 10:60326910 in GRCh37), an intronic variant in the *BICC1* gene

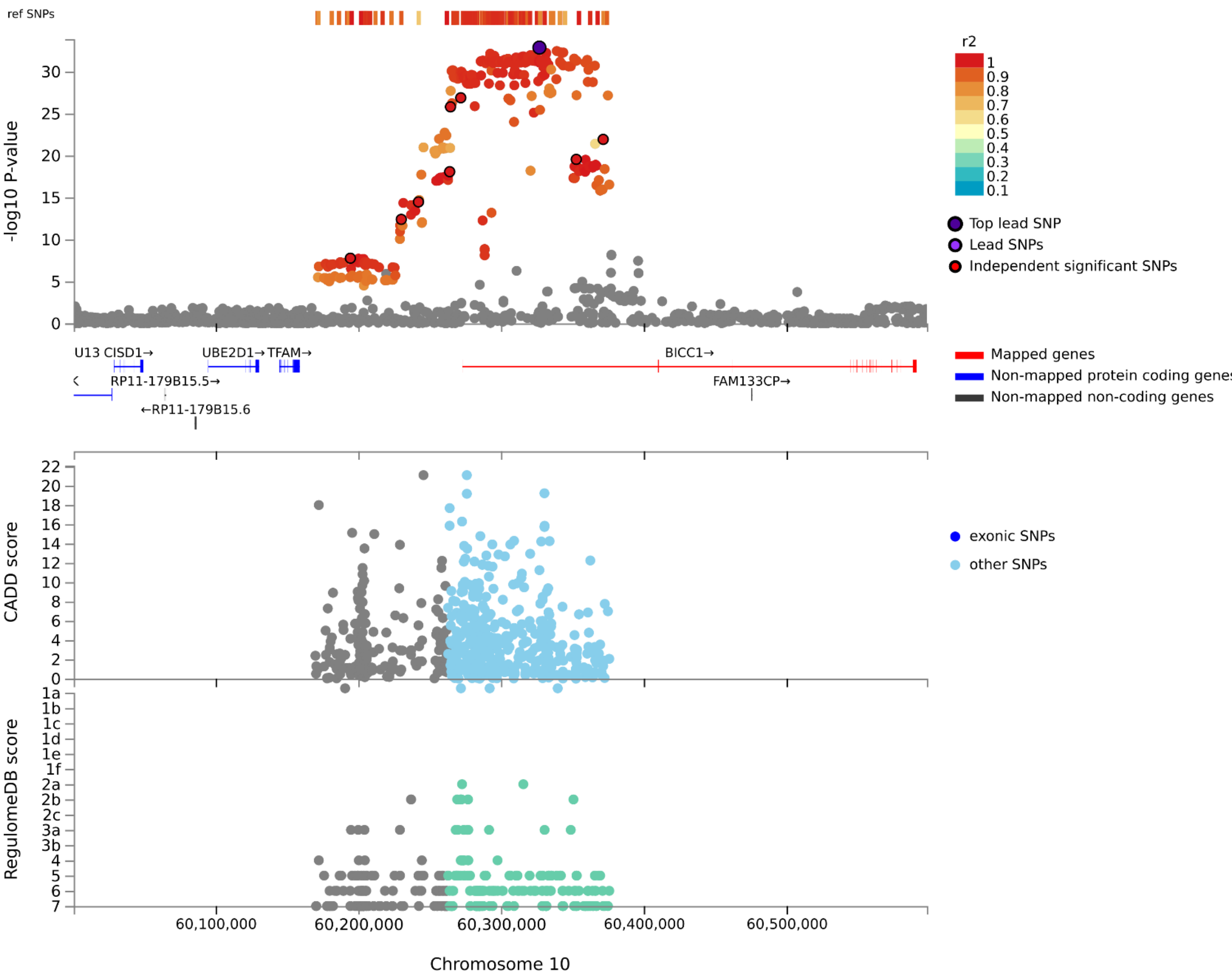

GRCh37 = Genome Reference Consortium human build 37, SNP = single nucleotide polymorphism, CADD = Combined Annotation Dependent Depletion, RegulomeDB = Regulome DataBase
