# Supplementary material for: A Comprehensive Assessment of the Shared Genetic Architecture between Myopia and Open-Angle Glaucoma
Source: Ophthalmol Sci. 2026 Jul 22;6(10):101328. doi: 10.1016/j.xops.2026.101328 (PMC13553571; doi:10.1016/j.xops.2026.101328)
Supplement: Supplementary file 9 [file mmc7.pdf]

Figure S8. Regional association plots of significant pleiotropic locus rs34935520 (i.e. 14:61091401 in GRCh37), an intergenic variant between the *SIX1* and *SIX6* genes

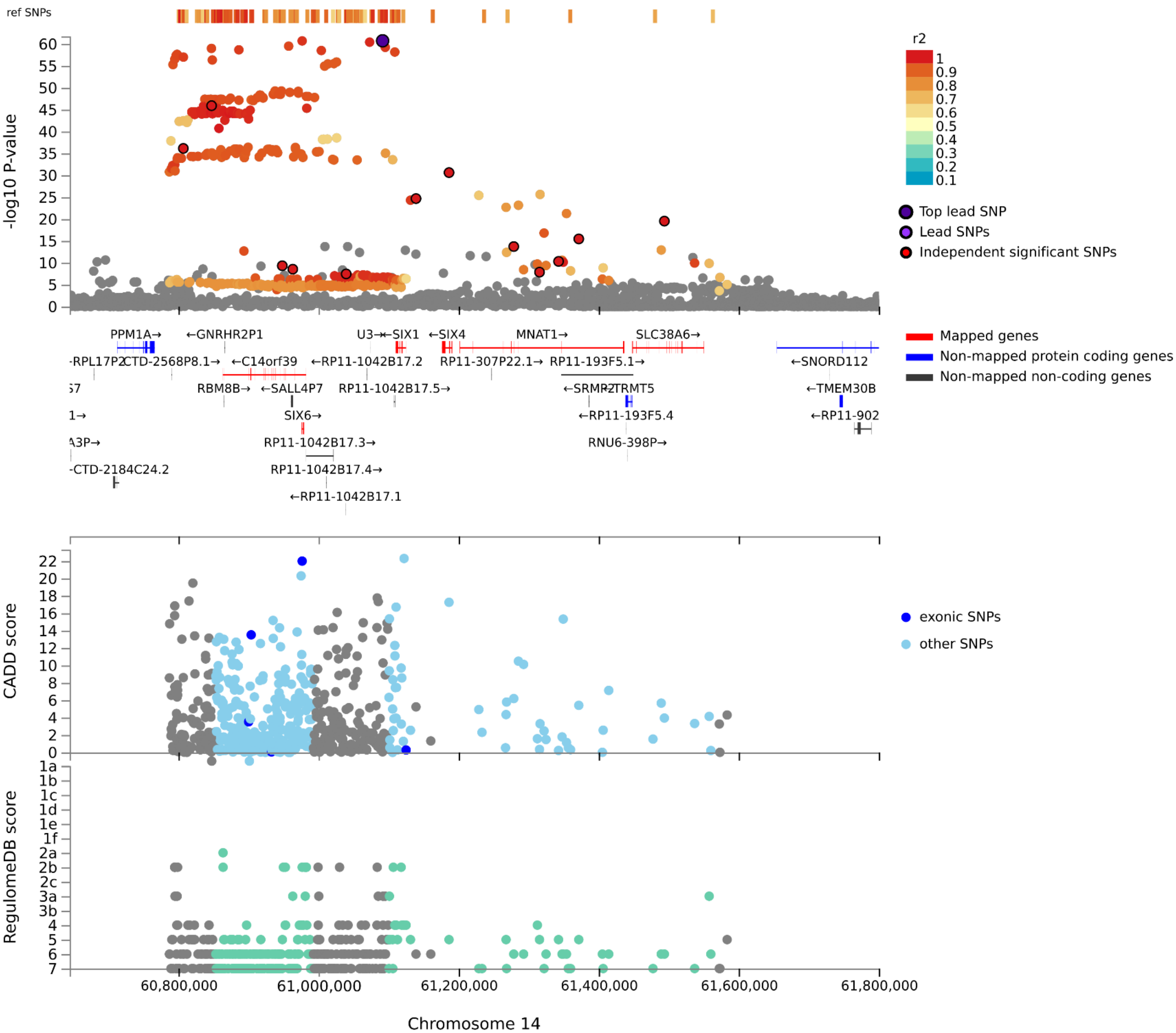

GRCh37 = Genome Reference Consortium human build 37, SNP = single nucleotide polymorphism, CADD = Combined Annotation Dependent Depletion, RegulomeDB = Regulome DataBase
