# Supplementary material for: A Comprehensive Assessment of the Shared Genetic Architecture between Myopia and Open-Angle Glaucoma
Source: Ophthalmol Sci. 2026 Jul 22;6(10):101328. doi: 10.1016/j.xops.2026.101328 (PMC13553571; doi:10.1016/j.xops.2026.101328)
Supplement: Supplementary file 10 [file mmc8.pdf]

Table S2. Overview of recruitment period, predominant ancestry, and study design of included studies.

**Study**	**EPIC-Norfolk Eye Study**	**Gutenberg Health Study**	**Rotterdam Study**	**Busselton Healthy Aging Study**	**The Raine Study Gen2**	**Beijing Eye Study**	**Dutch Myopia Study**
**Recruitment period**	1993-1997	2007-2012	1991-2006	2010-2015	1989-1991	2011	2010-2012
**Ancestry**	European	European	European	European	European	East Asian	European
**Definition of OAG**	Glaucoma specialist	ISGEO criteria	Visual field criteria	Self report	N/A	Optic disc assessment	Self report
**Measurements**							
**IOP**	NCT & Ocular Response Analyzer	NCT	Goldmann applanation & NCT	Rebound tonometry	Rebound tonometry	Goldmann applanation	Goldmann applanation
**VCDR**	HRT	Fundus photography	Fundus stereo photography & HRT	Fundus photography	OCT	Fundus photography	Fundus photography
**Genotyping array**	Affymetrix UK Biobank Axiom	Illumina Omni 2.5 (EURHD)	Infinium II HumanHap550 & 610-Quad BeadChip	Illumina 660W-Quad BeadChip	Illumina 660W-Quad BeadChip	Illumina 610K & Infinium OmniExpress	Illumina Omni Express
**Imputation panel**	HRC v1.0 & 1000G phase 3	1000G phase 3	TOPMed	TOPMed	TOPMed	1000G Asia Project	1000G phase 3

EPIC = European Prospective Investigation into Cancer and Nutrition, OAG = open-angle glaucoma, ISGEO = International Society for Geographical and Epidemiological Ophthalmology, N/A = not applicable, IOP = intraocular pressure, NCT = non-contact tonometry, VCDR = vertical cup-disc ratio, HRT = Heidelberg Retinal Tomograph, OCT = optical coherence tomography, HRC = Haplotype Reference Consortium, 1000G = 1000 Genomes, TOPMed = Trans-Omics for Precision Medicine
