# Supplementary material for: A Comprehensive Assessment of the Shared Genetic Architecture between Myopia and Open-Angle Glaucoma
Source: Ophthalmol Sci. 2026 Jul 22;6(10):101328. doi: 10.1016/j.xops.2026.101328 (PMC13553571; doi:10.1016/j.xops.2026.101328)
Supplement: Supplementary file 11 [file mmc9.pdf]

Table S3. Multivariable logistic regression of a standardized myopia GRS with high myopia and the AUC for the GRS alone, with corresponding 95% confidence intervals.

Study	N, controls	N, high-myopes (%)	OR / SD (95% CI)	AUC (95% CI)
**EPIC-Norfolk**	5,893	271 (4,40)	1.83 (1.60, 2.11)	0.69 (0.66, 0.72)
**Gutenberg Health Study**	11,747	642 (5.20)	1.77 (1.63, 1.91)	0.66 (0.64, 0.68)
**Rotterdam Study**	8,146	371 (4.36)	2.23 (1.98, 2.50)	0.68 (0.65, 0.71)
**Busselton Healthy Aging Study**	4,437	91 (2.00)	1.75 (1.41, 2.17)	0.66 (0.60, 0.72)
**The Raine Study Gen2**	1,072	25 (2.30)	2.71 (1.80, 4.10)	0.77 (0.67, 0.87)
**Beijing Eye Study**	1,115	39 (3.40)	1.09 (0.83, 1.44)	0.50 (0.42, 0.58)
**Dutch Myopia Study**	428	514 (54.56)	1.93 (1.65, 2.26)	0.67 (0.63, 0.71)

All associations were corrected for age and sex.
GRS = genetic risk score, AUC = area under the curve, OR = odds-ratio, SD = standard deviation, EPIC = European Prospective Investigation into Cancer and Nutrition

Table S4. Multivariable logistic regression of the standardized OAG GRS with OAG and the AUC for the GRS alone, with corresponding 95% confidence intervals.

Study	N, controls	N, OAG (%)	OR / SD (95% CI)	AUC (95% CI)
**EPIC-Norfolk**	5,947	217 (3.52)	1.75 (1.53, 2.01)	0.74 (0.71, 0.77)
**Gutenberg Health Study**	12,306	83 (0.80)	2.16 (1.74, 2.68)	0.71 (0.65, 0.77)
**Rotterdam Study**	8,265	252 (3.01)	1.77 (1.58, 1.98)	0.65 (0.62, 0.68)
**Busselton Healthy Aging Study**	4,478	84 (1.84)	1.64 (1.31, 2.04)	0.68 (0.62, 0.73)
**Beijing Eye Study**	1,119	35 (3.0)	1.49 (1.04, 2.13)	0.60 (0.50, 0.70)
**Dutch Myopia Study**	915	27 (2.87)	1.87 (1.27, 2.77)	0.66 (0.58, 0.76)

All associations were adjusted for age and sex.
OAG = open-angle glaucoma, GRS = genetic risk score, AUC = area under the curve, OR = odds-ratio, SD = standard deviation, EPIC = European Prospective Investigation into Cancer and Nutrition
